# The Postpartum Specific Anxiety Scale: development and preliminary validation

**DOI:** 10.1007/s00737-016-0658-9

**Published:** 2016-08-29

**Authors:** Victoria Fallon, Jason Christian Grovenor Halford, Kate Mary Bennett, Joanne Allison Harrold

**Affiliations:** Institute of Psychology, Health and Society, University of Liverpool, Eleanor Rathbone Building, Bedford Street South, Liverpool, L69 7ZA UK

**Keywords:** Postpartum, Anxiety, Maternal mental health, Scale development, Psychometrics

## Abstract

Perinatal symptoms of anxiety are increasingly recognised due to their high prevalence and impact. Studies using pregnancy-specific anxiety measures have found that they may predict perinatal outcomes more effectively than general measures. However, no such measure exists to assess anxieties specific to the postpartum. This study aimed to develop and validate a measure (Postpartum Specific Anxiety Scale; PSAS) that accurately represents the specific anxieties faced by postpartum women, using a four-stage methodology: (1) 51 items were generated from interviews conducted with a group of 19 postpartum women at two time points, (2) the scale was reviewed and refined by a diverse expert panel, (3) an online pilot study (*n* = 146) was conducted to assess comprehensibility and acceptability and (4) an online sample of 1282 mothers of infants up to 6 months old completed the PSAS against a battery of convergent measures. A subsample (*n* = 262) repeated the PSAS 2 weeks later. The PSAS possessed good face and content validity and was comprehensible and acceptable to postpartum women. PSAS scores were significantly correlated with other measures indicating good convergent validity. Principal component analyses (PCA) revealed a simple four-factor structure. Reliability of the overall scale and individual PSAS factors proved to be good to excellent. A preliminary receiver operating characteristic (ROC) analysis also suggested that the PSAS may be a useful screening tool. The psychometric evidence suggests that the PSAS is an acceptable, valid, and reliable research tool to assess anxieties, which are specific to the postpartum period. Next steps in the iterative validation process are considered for both research and screening purposes.

## Introduction

Up to 20 % of women in developed countries experience mental health problems postnatally (World Health Organisation [WHO] 2016). Several decades of research have focused on postpartum depression, while symptoms of anxiety have been largely overlooked. However, postpartum anxiety has become a condition of interest to perinatal researchers and practitioners in recognition of high prevalence rates and impact on maternal and infant outcomes (Lonstein 2007; Glasheen et al. 2010; Paul et al. 2013). Studies of postpartum anxiety reveal incidence estimates ranging from 3 to 43 %, with evidence suggesting that it may occur independently and at a higher rate than postpartum depression (PPD) does (Wenzel et al. 2005; Britton 2008; Glasheen et al. 2010; Paul et al. 2013).

The postpartum period involves a series of temporally unique transitions which are often experienced as stressful and overwhelming. This can lead to specific postpartum concerns such as personal appearance and postpartum weight gain (Walker and Freeland‐Graves 1998), health and wellbeing of the infant (Lugina et al. 2004), interpersonal relationships (Hiser 1991) and general infant care (Warren 2005). Comprehensive reviews by Lonstein (2007) and Glasheen et al. (2010) also link postpartum anxiety to a range of adverse developmental, somatic and psychological outcomes in the infant. The interpretation of these results, however, is limited by the use of general scales of anxiety such as the State Trait Anxiety Inventory (STAI) (Spielberger et al. 1970) and/or scales that focus predominantly on postpartum depression (i.e. the Edinburgh Postnatal Depression Scale; EPDS; Cox et al. 1987).

General measures of anxiety are relied upon in a large majority of studies examining postpartum anxiety^1^ (Glasheen et al. 2010; Lonstein 2007; Meades and Ayers 2011) and may be psychometrically problematic. Many commonly used general measures include somatic items which may occur naturally in the postpartum (e.g. STAI: ‘I feel rested’ or ‘I feel comfortable’). These may inflate anxiety scores in postpartum populations (Meades and Ayers 2011) and increase the likelihood of false positives (Swallow et al. 2003). Furthermore, symptoms of anxiety occurring in the postpartum may have distinct presentations which are not encompassed by items in general scales (Meades and Ayers 2011; Phillips et al. 2009); this limitation has been addressed when examining anxieties occurring in pregnancy (Van den Bergh 1990; Levin 1991; Wadwha et al. 1993; Huizink et al. 2002).

A variety of self-report questionnaires has been developed to assess specific anxieties relating to the gestational period that would not bear relevance in general scales. These include the Pregnancy Anxiety Scale (PAS; Levin 1991), the Pregnancy-Related Anxiety Questionnaire (PRAQ; Van Den Bergh 1990), the PRAQ-R (Huizink et al. 2004) and the Pregnancy-Related Anxiety Scale (PRAS; Wadwha et al. 1993). These measures include constructs such as fear of childbirth, foetal health and wellbeing, bearing a physically or mentally handicapped child, the mother-infant relationship, relationship changes and changes in appearance. Two key findings have been observed by studies using these measures: (a) that they predict perinatal outcomes more effectively than general measures of anxiety do (Wadwha et al. 1993; Sandman 1997) and (b) that they are qualitatively and quantitatively distinct from general indices of anxiety and depression (Huizink et al. 2002). This has led researchers to regard pregnancy-specific anxiety as a distinct entity to anxiety experienced at other times of life (Huizink et al. 2004).

In a similar manner, postpartum-specific scales have been designed to measure depression. These include the EPDS (Cox et al. 1987) and the Postpartum Depression Screening Scale (Beck and Gable 2000). Given high comorbidity with anxiety in some postpartum samples, some researchers have argued that they may be utilised to screen for both anxiety and depression concurrently (Stuart et al. 1998; Ross et al. 2003; Reck et al. 2008). While three items of the EPDS have been found to cluster together on an anxiety factor in postpartum women in several studies (Ross et al. 2003; Matthey 2008; Phillips et al. 2009; Matthey et al. 2013), the authors maintain that the scale does not measure anxiety (Cox et al. 1987). Furthermore, the EPDS does not distinguish whether anxiety scores on these three items are a feature of depression or a distinct entity (Matthey et al. 2003; Ross et al. 2003). This limits the utility of such tools considering work which finds that anxiety occurs more frequently (Muzik et al. 2000; Wenzel et al. 2005; Paul et al. 2013) and independently (Muzik et al. 2000; Matthey et al. 2003; Wenzel et al. 2005; Miller et al. 2006) of depression in the postpartum.

Two recent endeavours have been made to create an anxiety scale relevant to postpartum women: the Perinatal Anxiety Screening Scale (PASS; Somerville et al. 2014) and the Postpartum Worry Scale-Revised (PWS-R; Moran et al. 2014). Both measures aim to detect clinically significant levels of anxiety which map onto existing diagnostic criteria for anxiety disorders, although the PWS-R focuses only on generalised anxiety disorder (Moran et al. 2014). Emerging evidence highlights a large number of postpartum women who do not meet diagnostic criteria for an existing anxiety disorder yet experience a clinically significant degree of ‘maternally focused worry’ (Wenzel et al. 2005; Phillips et al. 2007; Phillips et al. 2009). As such, items within these scales may not encompass the full range of symptoms of anxiety experienced postnatally and a scale with a more focused domain is necessary. Furthermore, the PASS was designed for use with both antenatal and postnatal women (Somerville et al. 2014) suggesting that symptoms are comparable across childbirth. Although an overlap between pregnancy and postpartum anxiety has been identified (Heron et al. 2004), a body of literature provides evidence for a temporally specific pregnancy anxiety (Van den Bergh 1990; Levin 1991; Wadwha et al. 1993; Huizink et al. 2004), which includes constructs such as ‘fear of childbirth’ (Huizink et al. 2004) that would not be applicable postpartum. Furthermore, some women may be more prone to developing postpartum anxiety as consequence of specific physiological and psychological processes associated with birth (Heron et al. 2004), which raises additional considerations for measurement. Finally, items from both the PASS and the PWS-R were generated from researcher/clinician experience (Moran et al. 2014; Somerville et al. 2014). Although clinicians may be the best observers of the outward manifestations of symptoms or disorder, only those who experience it can effectively capture the subjective elements (Streiner et al. 2015). This multifactorial rationale supports the development of an anxiety scale specific to the postpartum period which takes into account the limitations of the existing evidence base.

## Research aims

1. To develop and validate a postpartum-specific anxiety scale
2. To investigate the structure of specific fears and worries related to the postpartum period (‘postpartum anxieties’) using this new scale

## PSAS development

The PSAS was developed by a doctoral student under the supervision of two experienced perinatal psychologists in the Department of Psychological Sciences at The University of Liverpool. All stages of the scale development and validation gained ethical approval from the University of Liverpool Institute of Psychology, Health and Society Ethics Committee in August 2015. All aspects of the study were performed in accordance with the 1964 Declaration of Helsinki.

## Stage 1: item generation

Items were predominately informed from interviews conducted with a group of 19 postpartum women at two time points (time one 4–8 weeks; time two 12–16 weeks) to ensure that an accurate, experiential representation of postpartum-specific anxieties was achieved. Responses to the open question ‘What are the main anxieties that women have at this stage of motherhood’ were digitally recorded, and a basic content analysis was performed to identify themes and develop items. The scale items were further developed by reviewing validated pregnancy and postpartum anxiety questionnaires (PASS: Somerville et al. 2014; PWS-R: Moran et al. 2014; PAS: Levin 1991; PRAQ: Van Den Bergh 1990; PRAQ-R: Huizink et al. 2004; PRAS: Wadwha et al. 1993) and the postpartum anxiety research literature. The item pool was developed to systematically encompass a broad range of anxieties that were temporally specific to the postpartum period.

Consistent with other validated scales in the field, the 51-item PSAS was formatted as a self-report questionnaire with a four-point Likert scale assessing the frequency of specific anxieties with consistent response options (from zero = ‘not at all’ to three = ‘almost always’). The order of 27 responses was randomly reversed in order to avoid ‘yea-saying’ bias and aid participant concentration (Streiner et al. 2015). The wording and amount of Likert-scale divisions were chosen based on best current practice in the psychometrics literature (Streiner et al. 2015) and careful review of the self-admitted limitations of already-validated anxiety scales (Somerville et al. 2014). The timeframe for rating frequency of anxieties was limited to over the past 7 days. This is congruent with pregnancy-specific anxiety scales and deemed necessary given the transient nature of anxieties occurring in the postpartum.

## Stage 2: expert panel and face and content validity

The preliminary 51-item scale was reviewed and refined by a panel of 12 individuals, each reflecting distinct insights of scale development and/or postpartum anxiety. The panel included three experienced perinatal researchers, three senior community midwives, three research midwives (one senior), one statistician and two psychometricians. Each panel member (blind to the other members’ feedback) provided detailed comments on individual items and the overall suitability of the scale. Qualitative responses from the panel indicated that the preliminary scale appeared to be measuring postpartum-specific anxieties and was both clinically acceptable for perinatal women and psychometrically feasible, indicating adequate face validity. The panel members also evaluated each item on a four-point Likert scale (four = highly relevant; three = quite relevant or highly relevant but needs rewording; two = somewhat relevant; and one = not relevant). A content validation ratio (CVR; Streiner et al. 2015) was calculated to provide a quantitative expression of content validity. The mean CVR across all items was 0.76 which is indicative of good content validity. The panel was also asked to comment on whether any items had been omitted to further establish content coverage.

Specific qualitative feedback was collated and analysis of this phase indicated a need to revise certain aspects of the scale. Thirty-two items were reworded based on the general consensus of the panel. Of particular importance was the rewording of 11 items to reflect the specificity of postpartum anxiety. For example, the item ‘I have worried about my relationship with my partner’ was reworded to ‘I have worried more about my relationship with my partner than before my baby was born’. Five items were deleted either due to repetition or because there was general agreement that they did not specifically relate to postpartum anxiety (e.g. low CVR). In addition, seven new items were included based on content coverage ideas provided by the panel.

The design and presentation of the final 53-item scale was then extensively reviewed to ensure it was streamlined and easy to respond to. The wording of the final items was subjected to a computer literacy check (Flesch-Kincaid Grade Level test) as being understandable for someone with 5 years of education or a 10-year-old child. A question understanding aid (QUAID) (Graesser et al. 2006) was also used, and no issues were found with the wording, syntax or semantics of the questions.

## Stage 3: pilot study

An online pilot study was conducted via the Qualtrics survey software platform to assess comprehensibility of item wording, ease of responding, time taken to complete and any other implementation issues. Mothers of infants aged between 0 and 6 months (*n* = 146) were recruited via online forums (Mumsnet, Netmums) and social media platforms (Facebook groups and Twitter). Participants completed the 53-item scale and rated comprehensibility and ease of responding on two 10-point Likert scales (i.e. ‘not at all easy to understand’ [0] to ‘extremely easy to understand’ [10] and ‘not at all easy to complete’ [0] to ‘extremely easy to complete’ [10]). An optional free text box was provided at the end of the survey to allow qualitative comments on the questionnaire content and experience of completion to be made.

The acceptability of the PSAS was excellent. The mean scores for the comprehensibility item and the ease of completion item were 9.29 (±1.24) and 9.18 (±1.26), respectively. The mean time taken to complete the 53-item scale was 9 min (range 3 to 15 min). Based on the qualitative responses from 18 women, a ‘not applicable’ option was created for 7 items relating to partner, families and work to avoid response ambiguity for women who may not have these life domains. Positive comments about the scale design and items were also recorded by 36 women, which provided further evidence of its acceptability in this population.

A preliminary item analysis (endorsement frequency and item homogeneity) was also conducted on the pilot study data. The overall scale had excellent reliability (Cronbach’s α = 0.96). Inter-item correlations were between 0.15 and 0.50. Item-total correlations were between 0.30 and 0.70. No problematic items were identified at this stage.

## Stage 4: scale reliability and validation study

### Method

#### Measures

##### Demographic information

Maternal demographic questions were asked at the beginning of the online survey, including maternal age, country of residence, marital status, skill level of occupation, educational attainment, current diagnosis of anxiety and depression, timing of diagnosis and any current antidepressant/anxiety medications. Infant demographic data was also asked, including infant age, birth order, multiple birth status (twins/triplets), timing of birth and mode of feeding.

##### The Edinburgh Postnatal Depression Scale (EPDS; Cox et al. 1987)

The EPDS is a 10-item self-report questionnaire administered to screen for depressive symptoms in the postnatal period. It is the most widely used and recommended screening scale for postnatal depression. Three items (items three, four and five) have been found to cluster together on an anxiety factor (EPDS-3A) to indicate postpartum anxiety (Matthey 2008; Matthey et al. 2013). Higher scores indicate higher levels of postpartum depressive symptoms with a score of over 10 (maximum score 30) indicating probable postpartum depression.

##### The Beck Depression Inventory-II (BDI-II; Beck et al. 1988)

The BDI is a widely used self-report instrument for detecting and measuring depression. It measures the severity of 21 symptoms of general depression experienced during the past 2 weeks with higher scores indicating more severe depressive symptoms. Twenty-five years of research literature provide evidence of its reliability and validity in clinical and non-clinical samples (Beck et al. 1988).

##### The Spielberger State-Trait Anxiety Inventory (STAI; Spielberger et al. 1970)

The STAI is a 40-item self-report questionnaire designed to measure general anxiety. It has two separate subscales to measure situational (state) and stable (trait) anxiety. The STAI is a reliable and valid measure used with clinical and non-clinical populations and more recently in perinatal samples (Meades and Ayers 2011; Spielberger et al. 1970). Higher scores on each four-point Likert scale item indicate higher levels of anxiety.

#### Participants

**Fig. 1:**
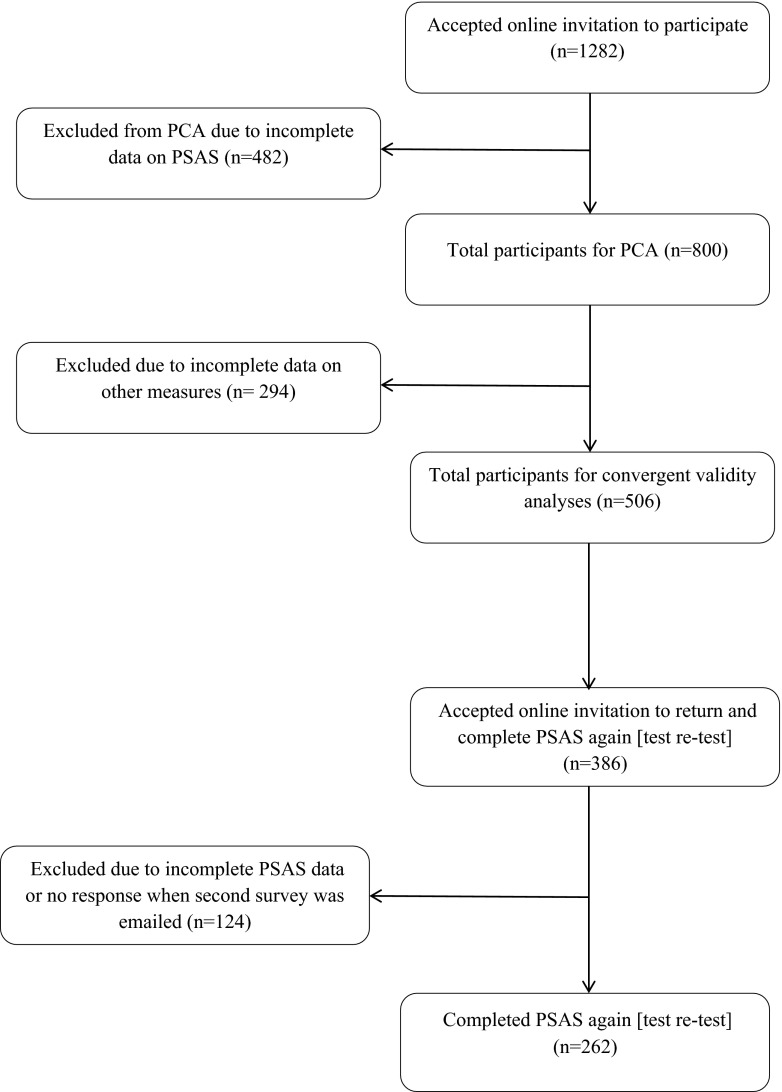
Participant flowchart

Participants were self-identified mothers (*n* = 1282) of infants aged between birth and 6 months postpartum. The 6-month cut-off point applied reflects the complete range of theorised postpartum phases (Romano et al. 2010). Of the 1282 participants, 482 (38 %) were excluded from the final analyses as they had missing data on the PSAS. For full details of participation rates at each stage of the study, see Fig. 1. The age of the final sample of 800 mothers ranged from 16 to 45 years (*M =* 30.78; *SD =* 4.96). The samples were predominately married (70 %), primiparous (50 %) and professional (40 %) women from the UK (84 %). One hundred fourteen (14 %) women had a current, clinical diagnosis of anxiety/depression at the time of participation, which is comparable with UK prevalence estimates. The babies’ ages ranged from 0 to 26 weeks (*M =* 16.20; *SD =* 7.08) (see Table 1 for full demographic details).

**Table 1:** Maternal and infant demographic characteristics (*n* = 800)

Maternal characteristic	Value	Infant characteristic	Value
Maternal age (mean years ± SD)	30.78 (±4.96)	Infant age (mean weeks ± SD)	16.20 (±7.08)
Country of residence (n/%)		Birth order (*n*/%)	
UK and Ireland	682 (85.2)	First	399 (49.9)
USA	63 (7.9)	Second	285 (35.6)
Australia and NZ	21 (2.7)	Third	85 (10.6)
Canada	10 (1.3)	Fourth	19 (2.4)
Other European	19 (2.3)	Fifth and after	12 (1.5)
Other non-European	5 (0.6)	Timing of birth (*n*/%)	
Marital status (*n*/%)		Premature (<37 weeks)	38 (4.7)
Married	563 (70.4)	Early term (>37 < 39)	156 (19.5)
Cohabiting	199 (24.9)	Full term (>39 < 41)	356 (44.5)
Single	32 (4)	Late term (>41 < 42)	141 (17.6)
Separated/divorced/widowed	6 (0.8)	Post term (>42 weeks)	109 (13.7)
Occupation (*n*/%)		Multiple birth (*n*/%)	
Managers, directors and senior officials	65 (8.1)	Yes	13 (1.6)
Professionals	319 (39.9)	No	787 (98.4)
Associate professional/technical	23 (2.9)	Mode of feeding (*n*/%)	
Administrative and secretarial	76 (9.5)	Exclusively breastfeeding	528 (66.0)
Skilled trades	14 (1.8)	Combination feeding	125 (15.7)
Caring, leisure and other service	91 (11.4)	Exclusively formula feeding	147 (18.4)
Sales and customer service	70 (8.8)		
Elementary occupations	4 (0.5)		
Housewife	114 (14.2)		
Not in paid occupation	24 (3.0)		
Educational attainment (*n*/%^a^)			
Postgraduate education	194 (24.3)		
Undergraduate education	313 (39.1)		
A-levels or equivalent college education	169 (21.1)		
GCSEs or equivalent secondary school education	83 (10.4)		
Other qualification	27 (3.4)		
No qualifications	14 (1.8)		
Current diagnosis of anxiety/depression (*n*/%)			
Yes	114 (14.2)		
No	680 (85.0)		
Prefer not to say	6 (0.8)		
Timing of diagnosis (*n*/%^a^)			
Before pregnancy	67 (58.8)		
During pregnancy	9 (1.1)		
Postpartum	38 (33.3)		
Currently prescribed medication for anxiety/depression diagnosis (N/%^a^)			
Yes	57 (50)		
No	57 (50)		

^a^Only participants who gave a ‘yes’ response to current diagnosis were included

#### Procedure

The participants were recruited through parenting forums (Mumsnet, Netmums), social media platforms (Facebook, Twitter) and other relevant websites via advertisements providing a link to the Qualtrics survey software. The advertisements stated that participants were invited to take part in a study to validate a new measure of postpartum anxiety. Prior to the main survey, an electronic consent form and information sheet were provided with a tick box to confirm that the main points had been read and understood. A single question enquired whether the participant was a mother to an infant aged between 0 and 6 months; only a positive response allowed entry to the main survey. Participants completed demographic questions followed by online versions of the PSAS, EPDS (including EPDS-3A), BDI and STAI (state and trait). On completion of all measures, the participants were invited to return 2 weeks later to complete the PSAS again as a measure of test-retest reliability for a reimbursement of £10. Those who were willing to return received an email with the second survey containing the PSAS 2 weeks later. Responses were linked via a unique ID embedded in the survey software to preserve anonymity. Online measurement provides greater convenience and anonymity than traditional paper-based methods do (Evans and Mathur 2005)^2^. The potential for repetitive responding was restricted via a ‘prevent ballot box stuffing’ option embedded in the survey software. The online survey was accessible from April 9, 2015 to May 11, 2015.

### Results

#### Factor structure of the PSAS

The factor structure of the PSAS was examined using data from all the participants who completed the scale (*n* = 800). A series of PCAs was conducted to determine the most appropriate number of factors to retain for rotation. Four factors were retained based on a combination of statistical tests: the results of the scree-test (Eigenvalues > 1 and the scree plot elbow point; Cattell 1966), cumulative variance explained (highest proportion of variance while retaining the simplest, most theoretical meaningful structure; Field 2009), parallel analysis (Eigenvalue that corresponds to the 95th percentile of the distribution of Eigenvalues derived from the random data; Glorfeld 1995) and MAP test (average partial correlations between the variables after successively removing the effect of the factors; O’Connor 2000). This model achieved the optimal structure but revealed that seven items had factor loadings below the 0.4 threshold. Five of these items were retained (‘I have felt that I should not need help to look after my baby’, ‘I have felt a greater need to do things in a certain way or order than before my baby was born’, ‘I have worried more about my finances than before my baby was born’, ‘I have felt that when I do get help, it is not beneficial’ and ‘I have worried that my baby is not developing as quickly as other babies’) based on sample size requirements for practical significance (Hair et al. 1979), adequate item-total correlations (>0.40), alpha if item deleted statistics (>0.95) and their theoretical relevance to postpartum anxiety, producing a 51-item scale. The PCA was conducted again, excluding the redundant items ‘I have felt under pressure from health professionals to care for my baby in a certain way’ and ‘I have had negative thoughts about my birth experience’. Sampling adequacy for the 51-item scale was excellent (KMO = 0.95) and Bartlett’s test of sphericity demonstrated that correlations between items were large enough for PCA (*χ* ^2^(1275) = 14,117.3, *p* < .001). The PCA revealed four factors, which in combination explained 44.72 % of the variance.

Theoretical review of the factor loadings was conducted by two authors (VF and JH) after oblique (direct oblimin) rotation (see Table 2). This revealed that factor 1 (competence and attachment anxieties) contained 15 items that addressed anxieties relating to maternal self-efficacy, parenting competence and the mother-infant relationship. Factor two (safety and welfare anxieties) had 11 items which were related to fears about infant illnesses, accidents and cot death. Factor three (practical baby care anxieties) included seven items covering anxieties that are specific to infant care such as feeding, sleeping and general routine. Finally, factor 4 (psychosocial adjustment to motherhood) contained 18 items which addressed adjustment concerns since the birth of the baby about management of personal appearance, relationships and support, work and finances and sleep.

**Table 2:** Factor structure of the PSAS (significant loadings in bold)

	Rotated components			
Scale item	1	2	3	4
Factor 1: maternal competence and attachment anxieties				
1.I have had negative thoughts about my relationship with my baby	**0.73**	−0.06	0.08	0.06
2.I have felt that my baby would be better cared for my someone else	**0.72**	0.01	0.03	−0.04
3.I have felt unconfident or incapable of meeting my baby’s basic care needs	**0.66**	0.10	0.20	−0.07
4.I have worried about the bond I have with my baby	**0.66**	0.050.	0.12	0.07
5.I have worried that my baby feels more content in someone else’s care	**0.62**	0.21	0.02	−0.05
6.I have felt that other mothers are coping with their babies better than me	**0.59**	−0.01	0.22	0.20
7.I have felt that I am not the parent I want to be	**0.57**	−0.04	−0.03	0.31
8.I have worried I will not know what to do when my baby cries	**0.54**	0.11	0.24	0.01
9.I have worried about how I will cope with my baby when others are not around to support me	**0.53**	0.09	0.08	0.08
10.I have worried about being unable to settle my baby	**0.52**	−0.05	0.36	0.02
11.I have worried that my baby is picking up on my anxieties	**0.49**	0.13	0.02	0.27
12.I have worried that my baby is less content than other babies	**0.47**	−0.05	0.42	−0.01
13.I have worried that other people think my parenting skills are inadequate	**0.41**	0.18	0.08	0.31
14.I have felt that motherhood is much harder than expected	**0.41**	−0.16	0.17	0.40
15.I have felt that I should not need help to look after my baby	**0.36**	0.09	−0.06	0.26
Factor 2: infant safety and welfare anxieties				
16.I have worried about my baby being accidentally harmed by someone or something else	0.12	**0.76**	−0.02	−0.01
17.I have repeatedly checked on my sleeping baby	−0.05	**0.71**	0.05	0.02
18.I have worried that my baby will stop breathing while sleeping	−0.02	**0.68**	0.11	−0.02
19.I have felt frightened when my baby is not with me	0.03	**0.67**	−0.09	0.19
20.I have worried about leaving my baby in a childcare setting	−0.12	**0.55**	0.03	0.28
21.I have worried about accidentally harming my baby	0.27	**0.52**	0.00	−0.07
22.I have thought of ways to avoid exposing my baby to germs	−0.12	**0.51**	0.17	0.02
23.I have not taken part in an everyday activity with my baby because I fear they may come to harm	0.29	**0.48**	−0.09	0.10
24.I have worried about my baby’s health even after reassurance from others	0.16	**0.48**	0.42	−0.02
25.I have worried that I will become too ill to care for my baby	0.30	**0.43**	0.08	0.02
26.I have felt a greater need to do things in a certain way or order than before my baby was born	0.02	**0.29**	0.13	0.28
Factor 3: practical infant care anxieties				
27.I have worried about my baby’s milk intake	−0.01	0.05	**0.74**	−0.04
28.I have worried about my baby’s weight	0.07	0.12	**0.68**	−0.12
29.I have worried about getting my baby into a routine	0.08	−0.09	**0.67**	0.14
30.I have worried about the way that I feed my baby	0.15	0.07	**0.62**	0.00
31.I have worried about the length of time that my baby sleeps	0.10	−0.18	**0.54**	0.26
32.I have used the internet for reassurance about my baby’s health	0.00	0.27	**0.44**	0.08
33.I have worried that my baby is not developing as quickly as other babies	0.25	0.19	**0.32**	0.05
Factor 4: psychosocial adjustment to motherhood				
34.I have felt resentment towards my partner	0.05	−0.09	0.04	**0.59**
35.I have felt tired even after a good amount of rest	0.07	0.05	−0.03	**0.58**
36.I have worried more about my relationship with my partner than before my baby was born	0.11	0.16	−0.07	**0.57**
37.I have worried that I am not going to get enough sleep	0.07	−0.23	0.23	**0.56**
38.I have worried that my partner finds me less attractive than before my baby was born	−0.13	0.16	0.11	**0.56**
39.I have worried more about my relationship with my family than before my baby was born	0.13	0.04	−0.12	**0.54**
40.I have worried more about my appearance than before my baby was born	−0.26	0.06	0.10	**0.55**
41.I have worried more about completing household chores than before my baby was born	0.03	0.02	0.23	**0.52**
42.I have had difficulty sleeping even when I have had the chance to	0.01	0.20	0.01	**0.51**
43.I have felt that I do not get enough support	0.26	0.01	−0.01	**0.49**
44.I have worried more about my relationship with my friends than before my baby was born	0.16	0.14	−0.05	**0.48**
45.I have been less able to concentrate on simple tasks than before my baby was born	0.25	0.07	−0.01	**0.47**
46.I have worried about returning to work	−0.18	0.27	0.07	**0.46**
47.I have felt unable to juggle motherhood with other responsibilities	0.38	−0.07	−0.13	**0.45**
48.I have felt that I have had less control over my day than before my baby was born	0.25	−0.09	0.20	**0.43**
49.I have felt isolated from family and friends	0.35	0.18	−0.12	**0.40**
50.I have worried more about my finances than before my baby was born	−0.11	0.22	0.17	**0.35**
51.I have felt that when I do get help it is not beneficial	0.25	0.27	0.01	**0.31**
% of variance explained	29.94	6.35	4.84	3.56
Cronbach’s alpha	0.91	0.85	0.80	0.90

Cross-loading items (i.e. items 14, 24, 26, 47, 49 and 51) were retained in the component with the highest loading and theoretical congruence to the other items in the factor. Item 14 (‘I have felt that motherhood is much harder than expected’) had similar loadings on factor 1 (competence and attachment anxieties) and factor 4 (psychosocial adjustment to motherhood). Though this item may represent difficulty adjusting, it is a competency-based question and was therefore retained in factor 1. Similarly, item 47 (‘I have felt unable to juggle motherhood with other responsibilities’) loaded onto factors 1 and 4. This item represented management of responsibilities and was better suited to factor 4. Items 24 (‘I have worried about my baby’s health even after reassurance from others’) and 26 (‘I have felt a greater need to do things in a certain way or order than before my baby was born’) reflect the obsessive-compulsive symptoms of anxiety that are often grounded in infant safety and welfare and were retained in factor 2. Items 49 (‘I have felt isolated from family and friends’ and 51 (‘I have felt that when I do get help it is not beneficial’) both represent management of support networks and were retained in factor 4.

The four factors had excellent reliability (Cronbach’s alpha ranged from 0.80 to 0.91; see Table 2) and had low to moderate correlations (*r* values ranged .26 to .39) indicating that they are not derived from a single underlying latent variable. The overall scale had excellent reliability (Cronbach’s α = 0.95).

#### Convergent validity of the PSAS

The participants who completed all convergent and divergent measures were included in this analysis (*n* = 506). The PSAS total score was significantly correlated with theoretically related measures of anxiety (i.e. EPDS-A, STAI-state and STAI-trait) and depression (i.e. EPDS, BDI) indicating good convergent validity (Table 3).

**Table 3:** Pearson product-moment correlations between the PSAS and other validated measures of anxiety and depression (*n* = 506)

	BDI	STAI-state	STAI-trait	EPDS	EPDS-A
PSAS	0.76*	0.74*	0.77*	0.81*	0.75*

*p < .01 (one tailed)

#### Preliminary screening accuracy of the PSAS

**Fig. 2:**
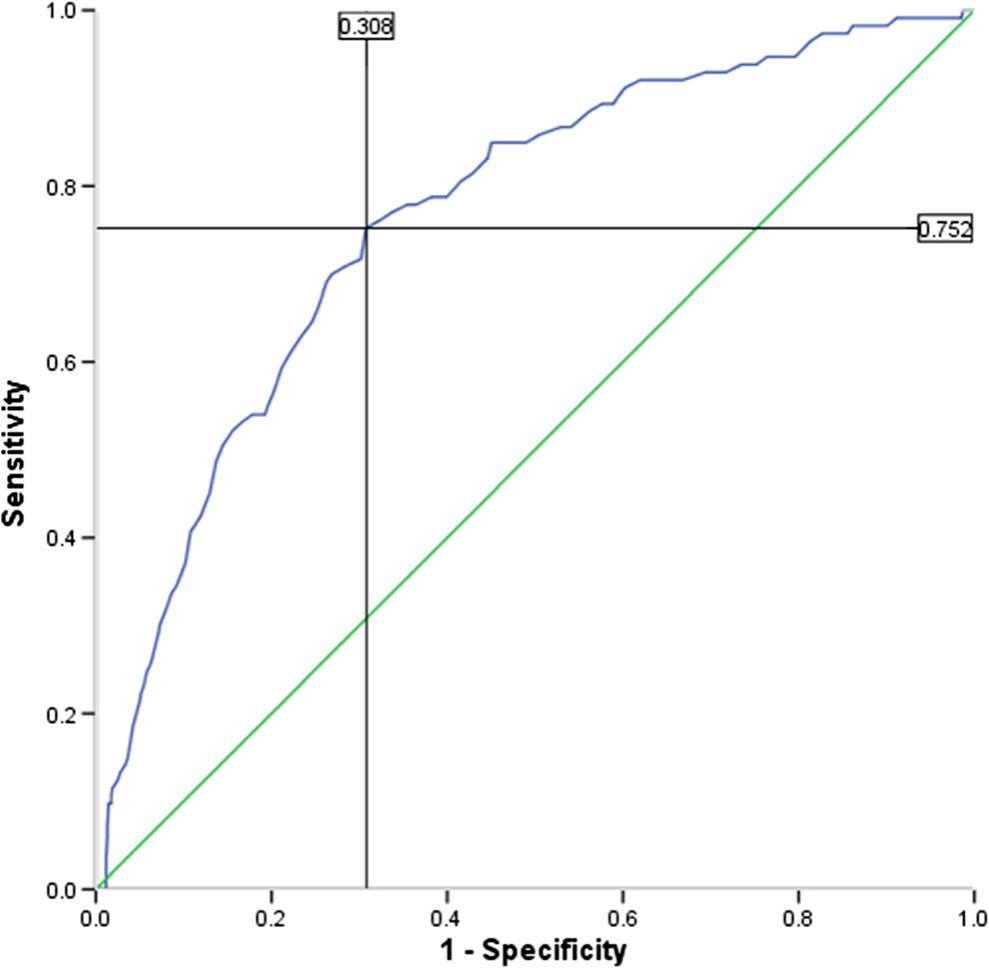
Receiver operating characteristic (ROC) curve analysis. Area under the curve 0.77

To preliminarily evaluate the performance of the PSAS in distinguishing between those with/without a current clinical diagnosis of anxiety/depression, a receiver operating characteristic (ROC) analysis was conducted. A statistically significant ROC curve (AUC 0.77; SE 0.02; *p* < .001; 95 % CI 0.72, 0.81; Fig. 2) revealed that the optimal cut-off PSAS score for detecting clinical levels of anxiety/depression was 112 with a sensitivity and specificity of 0.75 and 0.31, respectively. When compared to the recommended cut-off scores for the other included anxiety measures (STAI-S (45); STAI-T (45); EPDS-A (6)), the PSAS performed marginally better than the EPDS-A, which identified 73 % of cases, and better than the STAI-S, which detected 63 % of cases. However, it did not perform as well as the STAI-T which identified 86 % of cases.

#### Test-retest reliability of the PSAS

Pearson correlation coefficient was calculated to examine the test-retest reliability of the PSAS for a subsample of participants (*n* = 262) who repeated the PSAS 2 weeks after the initial administration. The test-retest reliability coefficient for the PSAS was 0.88 (*p* < .001*)*, indicating excellent stability over time in the first 6 months postpartum.

## Discussion

This study reports the development and initial validation of the PSAS, a 51-item measure of postpartum specific anxiety, using a large online sample of mothers in the first 6 months postpartum. The results suggest that the PSAS is an acceptable measure with sound psychometric properties. The low to moderate size correlations between factors indicated that they are not derived from a single underlying latent variable. It has a simple four-factor structure which showed good face and content validity and can be distinguished as (1) competence and attachment anxieties, (2) infant safety and welfare anxieties, (3) practical baby care anxieties and (4) psychosocial adjustment to motherhood.

Despite limited discussion about the qualitative nature of the symptoms of postpartum anxiety, these constructs are theoretically meaningful when examined in relation to some recent work. Brockington et al. (2006) found qualitative themes of ‘fear of cot death’, ‘fear of the criticism of mothering skills’ and ‘fear of disordered maternal attachment’ in a sample of 129 women referred to psychiatric services. Similar symptoms were also found in a recent interview study (Highet et al. 2014) alongside a theme of ‘adjustment difficulties’ which included anxieties relating to changes in appearance, daily activities and social roles. Phillips et al. (2009) investigated symptom presentations of postpartum women with an anxiety disorder not otherwise specified (ADNOS). They identified 65 % of women reporting anxieties in relation to infant health, safety and wellbeing; 53 % with anxieties concerning performance as a mother; and 18 % with anxieties relating to practical day-to-day care of the infant. This finding suggests that the PSAS, unlike existing measures, may possess constructs that are sensitive to postpartum women experiencing clinically significant maternally focused worry, yet failing to meet diagnostic criteria for an anxiety disorder (Phillips et al. 2009). Further examination of the construct validity of the PSAS is necessary to reexamine the proposed model and to provide further confirmation of these factors.

As hypothesised, the PSAS was significantly positively correlated with theoretically related measures of anxiety, which demonstrates initial evidence of convergent validity. The PSAS was also significantly associated with measures of depression, which was anticipated given the high comorbidity identified in previous work (Stuart et al. 1998; Ross et al. 2003; Reck et al. 2008) and provides further convergent support. It has been suggested that the overlap between depression and anxiety reflects the co-occurrence of phenomenologically distinct constructs (Beck 1976; Beck et al. 1979; Burns and Eidelson 1998). As such, Burns and Eidelson (1998) contend that any valid and reliable measure of anxiety and depression should correlate approximately at the 0.70 level; the PSAS exceeded this benchmark. In addition, the internal consistency of the overall PSAS scale and four factors was good to excellent (George and Mallery 2003; Ponterotto and Ruckdeschel 2007). Test-retest reliability also indicated better stability over time than other recent endeavours (Somerville et al. 2014).

A preliminary ROC analysis demonstrated that the PSAS performed well at identifying women with a current clinical diagnosis of anxiety and/or depression. At the optimal cut-off score of 112, 75 % of women with a diagnosis were detected, which surpasses other recent efforts (Somerville et al. 2014). Furthermore, the PSAS performed better than did other general (i.e. STAI-S) and postpartum-specific (i.e. EPDS-A) measures of anxiety. However, determining the case finding abilities of the PSAS was not a primary aim of the research and it is acknowledged that the self-report methods used to ascertain a current, clinical diagnosis of anxiety and/or depression in the sample are crude compared to other work (Somerville et al. 2014). Furthermore, the design precluded the differentiation of anxiety and depression within the sample. Interestingly, trait anxiety had the best case-finding abilities and previous work has suggested that the trait scale may examine depression, as well as anxiety (Bieling et al. 1998; Julian 2011), which could explain the high area under the curve (AUC) observed in this sample. Despite these limitations, the analysis suggests that the PSAS may be a useful screening tool for postpartum women and future work in clinical samples across the full spectrum of anxiety disorders is necessary to confirm this.

In the interim, the PSAS can be used to capture a range of anxieties relating to both mother and infant, which are specific to the postpartum period. Other potential avenues for research use include examining the prevalence of postpartum-specific anxiety and examining how this varies in different populations (e.g. those with high-risk pregnancies, mothers of premature infants, mothers who have experienced previous miscarriage or stillbirth). Administering the PSAS in samples of postpartum women with non-comorbid anxiety and depression will allow examination of whether the PSAS measures ‘pure’ anxiety and can differentiate anxiety from depression. A comparison of scores on the PSAS in women with ADNOS and other anxiety disorders (e.g. GAD, OCD) would be particularly interesting given recent findings concerning maternally focused worry in samples of postpartum women with ADNOS (Phillips et al. 2007; Phillips et al. 2009).

Validation of a measure is an iterative process and there are several areas for future work that are necessary to continue the development and evaluation of the PSAS. Firstly, the study used an online convenience sample which provided an appropriate sample size for the analyses conducted (in particular PCA) but lacked sampling control. The samples were predominately married, professional women from the UK. Thus, the psychometric properties of the PSAS may vary in other populations, and it will be important to replicate these findings in diverse samples, particularly those at risk of developing postpartum anxiety. Second, the pilot study demonstrated excellent acceptability to postpartum women in its current form, which probably reflects the qualitative inquiry used to inform its development. However, the item analyses (inter-item, item total) displayed psychometric potential for the development of a short form, which may increase its utility in both clinical and research settings.

Finally, the pregnancy anxiety literature provides findings that differentiate pregnancy-specific anxiety from general measures of anxiety and depression (Huizink et al. 2004) and highlights that temporally specific measures may be more efficacious at predicting perinatal outcomes than the more commonly used general measures (Wadwha et al. 1993; Huizink et al. 2002; Huizink et al. 2003). Further research should attempt to replicate this work with the PSAS. Isolation of child-bearing-related anxiety from symptoms of general anxiety and depression may allow clinicians and researchers to address issues of identification, prediction and prevention more precisely (Huizink et al. 2004). Associations between postpartum anxiety and maternal attachment (Mertesacker et al. 2004), infant feeding (Britton 2007; Paul et al. 2013) and infant temperament (Coplan et al. 2005) have been previously identified and warrant examination to ascertain the predictive value of the PSAS for maternal and infant outcomes and determine whether it may be a more effective predictor of perinatal outcomes than general measures of anxiety.

## Footnotes

1 For a comprehensive review of anxiety measures validated in perinatal populations, refer to Meades and Ayers (2011)

2 For a comprehensive review of the value of online survey methods, see Evans and Mathur (2005)
